# Involvement of IL-17 A/IL-17 Receptor A with Neutrophil Recruitment and the Severity of Coronary Arteritis in Kawasaki Disease

**DOI:** 10.1007/s10875-024-01673-1

**Published:** 2024-03-07

**Authors:** I-Chun Lin, Jau-Ling Suen, Shau-Ku Huang, Ming-Hui Chou, Hsuan-Chang Kuo, Mao-Hung Lo, Kuang-Che Kuo, Lin Wang

**Affiliations:** 1 Department of Pediatrics, Graduate Institute of Clinical Medical Sciences, College of Medicine, Chang Gung Memorial Hospital-Kaohsiung Medical Center, Chang Gung University, Kaohsiung, Taiwan; 2 Graduate Institute of Medicine, College of Medicine, Kaohsiung Medical University, Kaohsiung, Taiwan; 3 National Institute of Environmental Health Sciences, National Health Research Institutes, Miaoli, Taiwan; 4 Department of Medicine, Johns Hopkins University School of Medicine, Baltimore, MD USA; 5 Graduate Institute of Clinical Medical Sciences, College of Medicine, Chang Gung University, Taoyuan City, Taiwan; 6 Department of Pediatrics, Po-Jen Hospital, Kaohsiung, Taiwan

**Keywords:** Coronary Arteritis, interleukin-17A, interleukin-17 Receptor A, Kawasaki Disease, *Lactobacillus casei* cell-wall Extract, Neutrophil Recruitment

## Abstract

**Purpose:**

To assess the role of the interleukin (IL)-17 A/IL-17 receptor A (IL-17RA) in Kawasaki disease (KD)-related coronary arteritis (CA).

**Methods:**

In human study, the plasma levels of IL-17 A and coronary arteries were concurrently examined in acute KD patients. In vitro responses of human coronary endothelial cells to plasma stimulation were investigated with and without IL-17RA neutralization. A murine model of *Lactobacillus casei* cell-wall extract (LCWE)-induced CA using wild-type Balb/c and *Il17ra*-deficient mice were also inspected.

**Results:**

The plasma levels of IL-17 A were significantly higher in KD patients before intravenous immunoglobulin therapy, especially in those with coronary artery lesion. The pre-IVIG IL-17 A levels positively correlated with maximal z scores of coronary diameters and plasma-induced endothelial mRNA levels of chemokine (C-X-C motif) ligand-1, IL-8, and IL-17RA. IL-17RA blockade significantly reduced such endothelial upregulations of aforementioned three genes and inducible nitric oxide synthase, and neutrophil transmigration. IL-17RA expression was enhanced on peripheral blood mononuclear cells in pre-IVIG KD patients, and in the aortic rings and spleens of the LCWE-stimulated mice. LCWE-induced CA composed of dual-positive Ly6G- and IL-17 A-stained infiltrates. *Il17ra*-deficient mice showed reduced CA severity with the fewer number of neutrophils and lower early inducible nitric oxide synthase and chemokine (C-X-C motif) ligand-1 mRNA expressions than *Il17ra*^+/+^ littermates, and absent IL-17RA upregulation at aortic roots.

**Conclusion:**

IL-17 A/IL-17RA axis may play a role in mediating aortic neutrophil chemoattraction, thus contributory to the severity of CA in both humans and mice. These findings may help to develop a new therapeutic strategy toward ameliorating KD-related CA.

**Supplementary Information:**

The online version contains supplementary material available at 10.1007/s10875-024-01673-1.

## Introduction

Kawasaki disease (KD), one of the most common childhood vasculitis, is characterized by robust systemic immune responses, followed by the subsequent development of inflammatory coronary arteritis (CA) of unknown etiology [1–3]. KD-related CA leads to coronary artery ectasia, aneurysms, and stenosis and is the leading cause of acquired heart disease during childhood [4, 5]. Because of its persistent vascular remodeling and long-term morbidity [6–8], the mechanism underlying systemic immune activation and subsequent localized leukocyte infiltration and vascular destruction urgently require elucidation.

Interleukin (IL)-17 A, belonging to the major human IL-17 family, is a proinflammatory cytokine that induces the production of chemokines [e.g., chemokine (C-X-C motif) ligand (CXCL)-1, IL-8, and macrophage inflammatory protein-3α], activates endothelial cells, and further facilitates neutrophil and monocyte recruitment to the site of inflammation [9]. It has been reported to play a pathogenic role in various autoimmune diseases, such as rheumatoid arthritis [10], and psoriasis [11], and chronic inflammatory disorders [12]. Growing evidence suggests that IL-17 and T helper 17 cells may be involved in KD immunopathogenesis [13, 14], and atherosclerosis [12, 15]. However, the mechanism of IL-17 family underlying KD-related CA have not been fully elucidated [16].

Previously, we found that KD patients and *Lactobacillus casei* cell-wall extract (LCWE)-stimulated mice shared a similar toll-like receptor-2 enhancement on monocytes [17], and that macrophage dectin-1 and spleen tyrosine kinase participated in murine immune responses and LCWE-induced CA [18]. Indeed, spleen tyrosine kinase plays a vital role in mediating collaborative cytokine production by toll-like receptor-2 and dectin-1 [19], which further influences T helper 17 cell differentiation [20], and IL-17 A-induced chemokine production [21]. IL-17 A mediates various downstream signaling pathways [e.g., chemokine (C-C motif) ligands (CCLs), and chemokine (C-X-C motif) ligands (CXCLs)] depending on cell and tissue types in different diseases or situations [22]. Therefore, we aimed to elucidate whether the IL-17 A signaling pathway might play a certain role in the local coronary inflammation in KD in this study.

## Materials and Methods

### Study Subjects and Samples

The KD patients who met the diagnostic criteria of typical or incomplete KD [3, 23, 24], and admitted at Kaohsiung Chang Gung Memorial Hospital, Taiwan, during 2013 and 2019 were recruited. Standard treatment with continuous 12-hour administration of intravenous immunoglobulin (IVIG, 2 g/kg/day) was initiated for acute KD patients with at least a 5-day fever. Blood samples were obtained within 24 h prior to the first IVIG (pre-IVIG), and 3 days and 1 month after IVIG. The plasma levels of IL-17 A were measured using a commercially available Human Cytokine/Chemokine Milliplex™ MAP kit (Millipore), and then detected using a Luminex^100^ Flowmetrix system (Luminex Corp.) as previously prescribed [18]. Meanwhile, some inpatients with fever lasting longer than 3 days with at least one of KD-suspicious symptoms were recruited as febrile controls (FCs).

### Echocardiography Study

Using Philips IE33 and EPIQ 7-α ultrasound machines, serial echocardiographic studies were performed simultaneously at time points of blood sampling. The body surface area-normalizing standard size (z-score) of each coronary segment was calculated using the Japan Kobayashi z-score calculator [25], and Taiwanese children reference [26]. The severity of coronary artery lesion (CAL) was minimally modified according to the clinical guidelines of the American Heart Association and Japanese Circulatory Society [3, 23], and graded from 1 to 5 using the maximal z-score of any coronary artery segment within 1 month from the onset.

### Coronary Artery Endothelial Cell Culture

Human coronary endothelial cells (HCAECs, Lonza) were cultured using the EGM-2 MV Microvascular Endothelial Cell Growth Medium-2 Bullet Kit (Lonza) at 37 °C with 5% CO_2_, and seeded in 24-well plates at a density of 2.5 × 10^4^ cells/well overnight for the stimulation experiment (15% pre-IVIG plasma for 4 and 24 h). For inhibition experiments, cells were pretreated with recombinant neutralizing antibody against human IL-17RA (10 µg/ml, no. MAB177; R&D) for 1 h and then cultured for a further 24 h.

### Transendothelial Neutrophil Migration

At first, neutrophils were first purified from the whole blood of healthy subjects as previously prescribed [27]. The cell morphology and the purity of neutrophils (> 98%) was confirmed for use. HCAECs (1 × 10^4^ cells) were seeded on a 24-well PET cell culture insert (3 μm pore membrane) for 4 hours, and then treated with pre-IVIG or FC plasma with or without neutralizing antibody to IL-17RA for another 24 h. The CellTracker Green (no. C2925, Invitrogen) labeled neutrophils (1 × 10^5^/µl, 100 µl) were then added to the top of each inserts and all were allowed to migrate for 5 hours. The amount of transendothelial migrated neutrophils were determined using fluorescence microscopy and counting cells with ImageJ.

### Flow Cytometry Analysis

To examine the surface expression of IL-17RA, the cells were separately stained with PE mouse anti-human IL-17RA (no. 566736, BD Biosciences) and PE mouse IgG1, κ isotype control (no. 554680; BD Biosciences). After staining, the cells were washed and resuspended in 200 microL PBS containing 2% paraformaldehyde for flow cytometry analysis using an LSRII Flow Cytometer (BD Biosciences).

### Mice and a Murine Model of KD-mimicry CA

Wild-type male BALB/c mice, *Il17 receptor a (ra)*-deficient mice (*Il17ra*^*−/−*^ on a C57BL/6 genetic background), and *Il17ra*^*+/+*^ littermates were used in this study. First-generation hybrid animals originating from an *Il17ra*^*−/−*^ and C57BL/6 cross were purchased from the National Laboratory Animal Center, Taiwan. LCWE preparation and LCWE-induced CA using 4–5-week-old male mice was performed as previously described [17, 18]. Mice were then sacrificed at indicated time points after induction, and cardiac tissues around aortic roots and spleens were extracted for further experiments.

### Histopathology, Immunohistochemistry, and Immunofluorescence

Cardiac tissue with surrounding aortic roots was meticulously extracted, perfused with ice-cold PBS, fixed in formalin, and embedded in paraffin. Serial 4-µm-thick slices of basal myocardium containing proximal coronary arteries and aortic roots were stained with hematoxylin and eosin to assess the severity of CA, which was blinded evaluated by a pathologist on a scale of 0 to 4 based on the number of cardiac infiltrates and the degree of fibrosis and myocardial destruction by histopathology, as described previously [18].

Immunohistochemistry staining was performed with a mouse monoclonal antibody for T cell marker (CD3, no. ab16669; Abcam), rat monoclonal antibodies for macrophage marker (F4/80, no. MCA497G; Bio-Rad), and neutrophil marker (Ly6G, no. sc-53,515; Santa Cruz Biotechnology), a rabbit polyclonal antibody for IL-17RA (no. ab218249; Abcam), and respective isotype control antibodies at 4 °C overnight and detected using a secondary antibody with the help of UltraVision Quanto Detection System HRP DAB Kit (Thermo Fisher Scientific) for CD3 and IL-17RA. Biotin goat anti-Rat with streptavidin HRP (BD Biosciences) for F4/80 and Ly6G were used. The number of IL-17RA-positive stained cells in spleens was the average obtained by counting five random 0.5 mm^2^ (0.5 mm x 1 mm) fields.

For immunofluorescence, sections were washed after incubation with primary antibodies of CD3, Ly6G, and IL-17 A (goat polyclonal antibody, no. sc-6077; Santa Cruz Biotechnology), overnight at 4 °C, and then followed by Alexa Fluor 488-conjugated goat anti-rat IgG (no. ab150157; Abcam) for CD3 and Ly6G, and Alexa Fluor 594-conjugated donkey anti-goat IgG (no. A-11058; Thermo) for IL-17 A for 60 min, respectively. After staining, the sections were mounted under coverslips using ProLong Gold antifade reagent with DAPI (Invitrogen). Slides were visualized using an Olympus FV10i confocal microscope (Olympus).

### RNA Isolation and Real-Time RT-PCR

Cultured HCAECs, human peripheral mononuclear cells, and murine aortic roots were homogenized, isolated, and reverse-transcribed to cDNA using previously prescribed methods [18]. Sequences of the paired primers are listed in Table S1. Human mRNA expression levels were expressed as a ratio in relation to the FC levels. Validations were performed in duplicate, and the amplification efficiencies were validated.

### Library Preparation and Sequencing

The purified RNA from aortic roots was used to prepare the sequencing library using the TruSeq Stranded mRNA Library Prep Kit (Illumina), following the manufacturer’s recommendations. Briefly, mRNA was purified from total RNA (1 µg) using oligo (dT)-coupled magnetic beads and was fragmented into small pieces at elevated temperatures. First-strand cDNA was synthesized using reverse transcriptase and random primers. After the generation of double-strand cDNA and adenylation on 30 ends of DNA fragments, the adaptors were ligated and purified with the AMPure XP system (Beckman Coulter). The quality of the libraries was assessed using an Agilent Bioanalyzer 2100 system and a real-time PCR system. The qualified libraries were then sequenced on an Illumina NovaSeq 6000 platform with 150 bp paired-end reads generated by Genomics, BioSci & Tech Co., New Taipei City, Taiwan.

### Bioinformatics Analysis

The bases with low quality and sequences from adapters in the raw data were removed using Trimmomatic (version 0.39) [28]. The filtered reads were aligned to the reference genomes using Bowtie2 (version 2.3.4.1) [29]. The user-friendly software RSEM (version 1.2.28) was used to quantify the transcript abundance [30]. Differentially expressed genes were identified using EBSeq (version 1.16.0) [31]. The fragments per kilobase per million values for genes were displayed using a heatmap created using ImageGP.

### Statistics

Data are expressed as median (interquartile range, IQR) and the number with the proportion of the number. Univariate analysis for the comparison of continuous variables was performed using the Mann–Whitney U-test and Kruskal–Wallis test. Correlation analysis was performed using Spearman’s correlation coefficient. The analysis of the human KD group was performed using generalized estimating equations for paired samples and data, and Wilcoxon signed-rank test for pair-comparisons of in vitro inhibition experiment. The chi-square test or Fisher’s exact test was used to compare the categorical variables. A *p*-value of < 0.05, determined using SPSS version 13.0 (SPSS Inc.), was considered statistically significant.

## Results

### Enlarged Coronary Segments in Early KD

In the human study, 68 patients with acute KD and 50 FCs were recruited (Table 1). The KD patients were younger and thus had smaller body weight, height, and BSA than FCs. Meanwhile, they had significantly larger numbers of peripheral white blood cells and platelets, higher percentages of segments, higher levels of C-reactive protein, fewer red blood cells, fewer percentages of lymphocytes and lower hemoglobin levels.

**Table 1:** Demography and clinical characteristics of the studied subjects

	FC (*n* = 50)	KD (*n* = 68)	*P*
Age, month-old	20.00 (11.00–58.00)	11.50 (6.00–21.75)	*0.001*
Male gender, *n* (%)	31 (62.00)	42 (61.76)	*0.979*
BW, kg	10.90 (8.60–14.50)	9.20 (7.33–11.70)	*0.013*
BH, cm	86.00 (72.20–100.25)	74.50 (66.00–85.05)	*0.005*
BSA, kg/m2	0.51 (0.42–0.62)	0.44 (0.38–0.53)	*0.008*
Laboratory test			
White blood cells, 10^3^/µL	9.80 (7.20–12.20)	13.70 (10.00–16.58)	*< 0.001*
Segment, %	42.10 (29.45 − 56.00)	59.00 (48.55–69.75)	*< 0.001*
Lymphocyte, %	45.10 (33.15–56.50)	29.25 (19.25–39.75)	*< 0.001*
Monocyte, %	6.50 (3.20–9.75)	6.00 (5.00–7.00)	*0.464*
Eosinophil, %	2.00 (0.15–4.70)	2.95 (1.00–4.70)	*0.056*
Red blood cells, 10^6^/µL	4.49 (4.28–4.83)	4.27 (3.95–4.56)	*0.012*
Hemoglobin, g/dL	11.90 (11.15–12.50)	11.00 (10.10–11.60)	*< 0.001*
MCV	78.20 (76.70–80.40)	77.40 (74.47–79.55)	*0.145*
Platelet, 10^3^/µL	253.00 (206.50–401.50)	356.50 (269.75–447.75)	*0.004*
CRP, mg/L	11.85 (3.48–38.85)	79.85 (28.95–128.20)	*< 0.001*
Incomplete KD, *n* (%)	NA	7 (10.29)	
IVIG resistance, *n* (%)	NA	10 (14.71)	
2nd IVIG, *n* (%)	NA	10 (14.71)	
Steroid therapy, *n* (%)	NA	6 (8.82)	
Coronary artery diameter, baseline			
LCA, mm	2.44 (2.23–2.86)	2.45 (2.10–2.94)	*0.987*
LCA z score	0.51 (0.12–1.20)	2.11 (1.25–3.01)	*< 0.001*
LAD, mm	2.04 (1.62–2.37)	2.03 (1.59–2.49)	*0.967*
LAD z score	0.63 (-0.31–0.94)	1.79 (1.13–2.71)	*< 0.001*
RCA, mm	2.00 (2.39–1.68)	1.88 (1.54–2.27)	*0.407*
RCA z score	0.29 (-0.50–0.84)	1.03 (0.49–2.71)	*< 0.001*
Coronary artery lesion,^a, b^*n* (%)	0		
z score < 2.5^c^	NA	21 (30.88)^a^/37 (53.62)^b^	
z score ≥ 2.5 and < 3^d^	NA	16 (23.53)^a^/11 (15.94)^b^	
z score ≥ 3 and < 5^e^	NA	25 (38.24)^a^/14 (20.29)^b^	
z score ≥ 5 and < 10^f^	NA	3 (4.41)^a^/6 (8.70)^b^	
z score ≥ 10, or diameter > 8 mm^g^	NA	3 (4.41)^a^/0^b^	

^a^Coronary artery lesion depends on z-scores of coronary diameters determined by Kobayashi z-score calculator

^b^Coronary artery lesion depends on z-scores of coronary diameters determined by Taiwanese z-score children reference

^c^Normal diameter of coronary artery defined as coronary artery lesion (CAL) grade 1

^d^Transiently mild dilatation of coronary arteries as CAL grade 2

^e^Small aneurysm as CAL grade 3

^f^Moderate aneurysm as CAL grade 4

^g^Large aneurysm as CAL grade 5

All data are expressed as median (interquartile, IQR) and the number with the portion of the number (%). BSA, body surface area; CRP, C-reactive protein; FC, febrile control; IVIG, intravenous immunoglobulin; KD, Kawasaki disease; LAD, left anterior descending artery; LCA, left main coronary artery; MCV, mean corpuscular volume; NA, non applicable; RCA, right coronary artery

Among KD patients, approximately one-tenth were incomplete type, one-seventh had intravenous immunoglobulin (IVIG) resistance and received a second IVIG, and one-twelfth received additional steroid therapy. Compared to FCs, KD patients had significantly greater pre-IVIG z-scores of coronary arterial diameters, which reduced after IVIG therapy (Figure S1). The pre-IVIG CRP level was positively correlated with their concurrent maximal coronary z-score (Spearman correlation, *r* = 0.28, *p* = 0.018) in KD patients. According to the Japan Kobayashi z-score calculator, approximately one-quarter of KD patients had transiently mild dilatation of coronary arteries, 38% had small aneurysms, 4% had moderate aneurysms, and 4% had large aneurysms within 1 month from the onset; whereas, using the Taiwanese z-score children reference, one-fifth had small aneurysms, and less than 10% had moderate aneurysms. None of the patients had a large aneurysm.

### Higher pre-IVIG Plasma Levels of IL-17 A in KD Patients with CAL

**Fig. 1:**
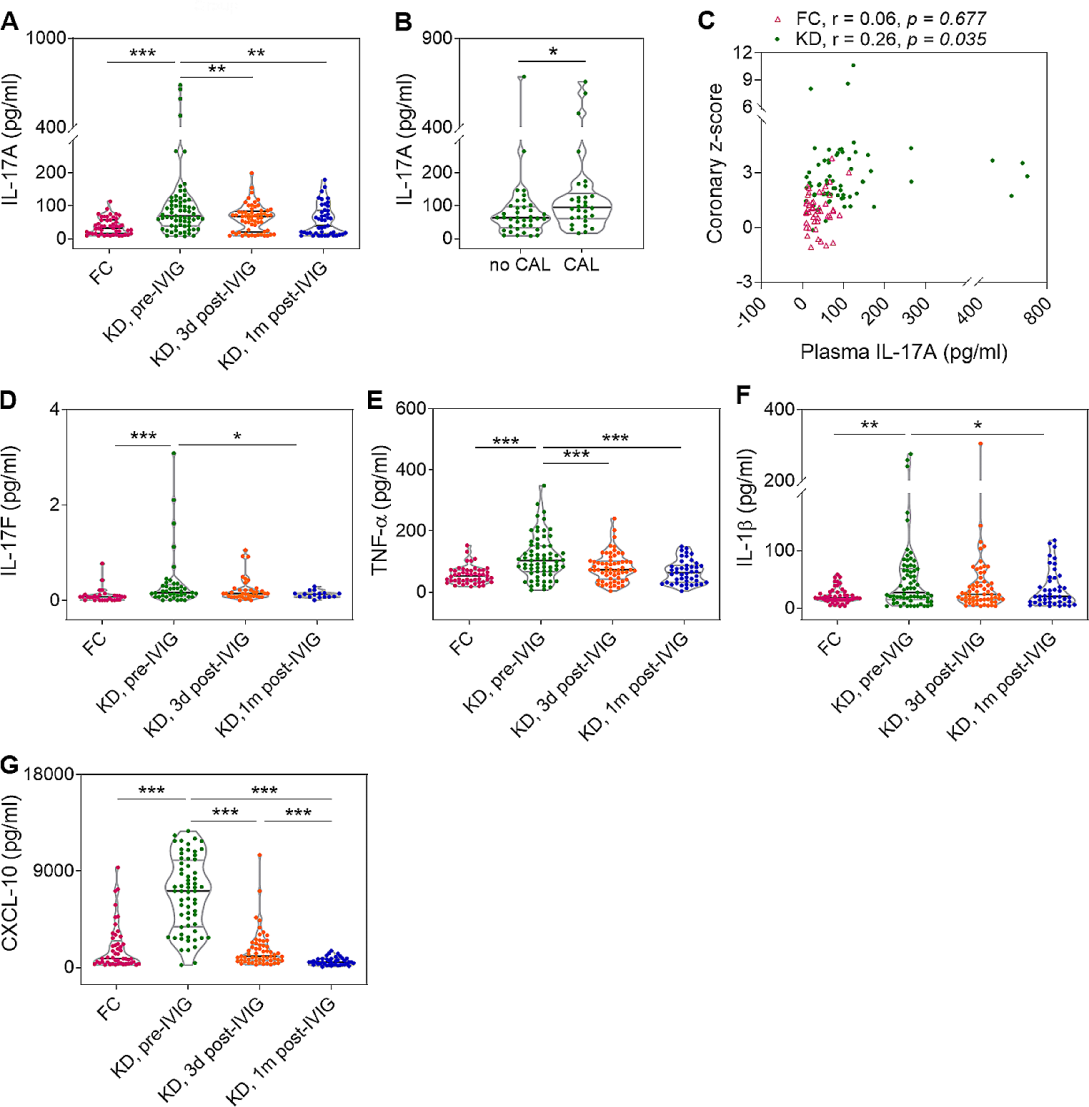
The plasma levels of IL-17 A and other cytokines in patients with acute Kawasaki disease (KD). **(A)** The pre-IVIG plasma level of IL-17 A was significantly higher than FC patients, which significantly reduced after IVIG therapy. **(B)** KD patients with CAL had significantly higher pre-IVIG plasma IL-17 A level than those without CAL. **(C)** Positive correlation of maximal coronary z-scores with the pre-IVIG plasma levels of IL-17 A. **(D-G)** The pre-IVIG plasma levels of IL-17 F, TNF-α, IL-1β, and CXCL-10 were significantly higher than FCs, which reduced after IVIG therapy. Horizontal lines on violin plots indicate median with interquartile ranges (IQRs).*P < 0.05, **P < 0.01, ***P < 0.001. CAL, coronary artery lesion; FC, febrile control; IVIG, intravenous immunoglobulin; Pre-IVIG, and 3d and 1 m post-IVIG, before, 3 days and 1 month after IVIG therapy.

The KD patients had significantly higher pre-IVIG plasma levels of IL-17 A (median, 74.50 pg/ml; IQR, 40.20–116.75 pg/ml) than the FCs (median, 32.45 pg/ml; IQR, 15.95–57.13 pg/ml; *p* < 0.001), which significantly reduced after IVIG therapy (Fig. 1A). Meanwhile, KD patients with CAL (grades 2–5) had significantly higher pre-IVIG plasma IL-17 A level (median, 95.21 pg/ml; IQR, 62.70–129.42 pg/ml) than those without CAL (grade 1) (median, 63.32 pg/ml; IQR, 33.23–97.68 pg/ml; *p* = 0.018, Fig. 1B). Of note, the pre-IVIG plasma levels of IL-17 A were positively correlated with the concurrently measured maximal coronary z-scores of coronary arteries although the correlation degree was weak (Fig. 1C). In addition, our KD patients had significantly higher pre-IVIG levels of IL-17 F, TNF-α, IL-1β, and CXCL-10 than FCs, which reduced after IVIG therapy (Fig. 1, D-G).

### Pre-IVIG KD Plasma Induced Endothelial IL-17RA-mediated Downstream CXCL-1 and IL-8 mRNA Productions and Neutrophil Transmigration

**Fig. 2:**
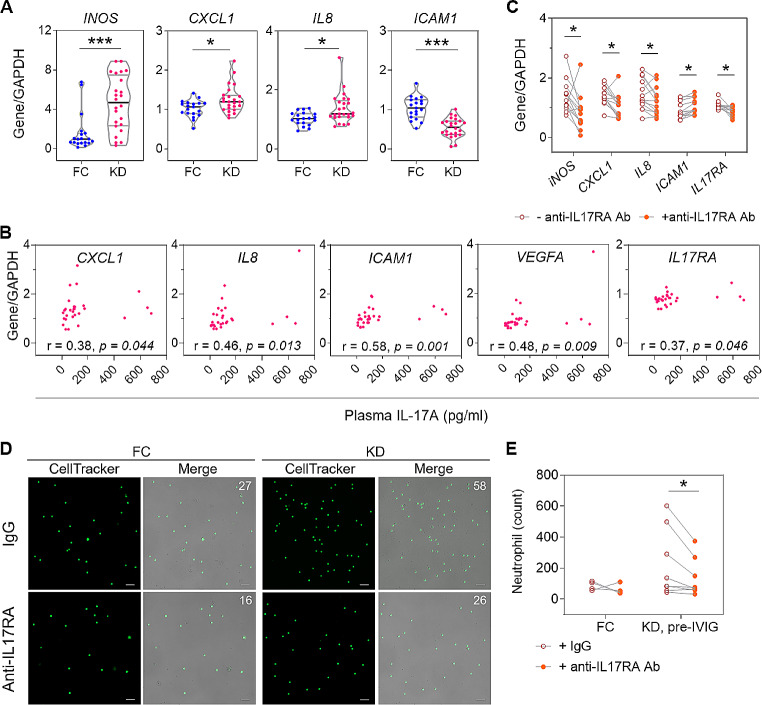
In vitro endothelial responses to stimulation with plasma from KD patients. **(A)** The mRNA expression levels of *INOS, CXCL1, IL8*, and *ICAM1* in KD (pre-IVIG) plasma- and FC plasma-treated HCAECs. **(B)** The correlation of endothelial mRNA expression levels of *CXCL1, IL8, ICAM1, VEGFA*, and *IL17RA* with the plasma levels of IL-17 A after 4 h-stimulation. **(C)** The HCAEC mRNA expression levels of *INOS, CXCL1, IL8, ICAM1*, and *IL17RA* in response to 24-hour plasma treatment with and without neutralizing anti-IL-17RA antibody. **(D)** The representative microphotographs show transmigrated neutrophils through plasma-stimulated HCAECs with neutralizing IL-17RA antibody or with IgG isotype (the number of migrated neutrophils shown on the right upper corner of each photo; scale bar: 100 μm), and **(E)** the quantitative analysis. Horizontal lines on violin plots indicate median with IQRs.*P < 0.05, **P < 0.01, ***P < 0.001. CXCL-1, chemokine (C-X-C motif) ligand-1; HCAEC, human coronary arterial endothelial cell; ICAM-1, intercellular adhesion molecule-1; IL-8, interleukin-8; iNOS; inducible nitric oxide synthase; VEGF-A, vascular endothelial growth factor-A

In the in vitro experiment, the pre-IVIG plasma induced significantly higher endothelial expression levels of inducible nitric oxide synthase (*INOS*), *CXCL1*, and *IL8*, and significantly lower intercellular adhesion molecule (*ICAM)-1* mRNA level than FC plasma after 24-hour stimulation (Fig. 2A). Interestingly, after 4-hour stimulation, the pre-IVIG plasma-induced endothelial mRNA levels of *CXCL1, IL8, ICAM1*, vascular endothelial growth factor-A, and *IL17RA* were positively correlated with the plasma levels of IL-17 A (Fig. 2B). The neutralizing anti-IL-17RA antibody significantly reversed the plasma-induced mRNA alterations of *INOS, CXCL1, IL8, ICAM1, and IL17RA* (Fig. 2C), and reduced the number of plasma-induced transendothelial migrated neutrophils (Fig. 2, D and E).

### IL-17RA Augmentation in Human KD and LCWE-injected mice

**Fig. 3:**
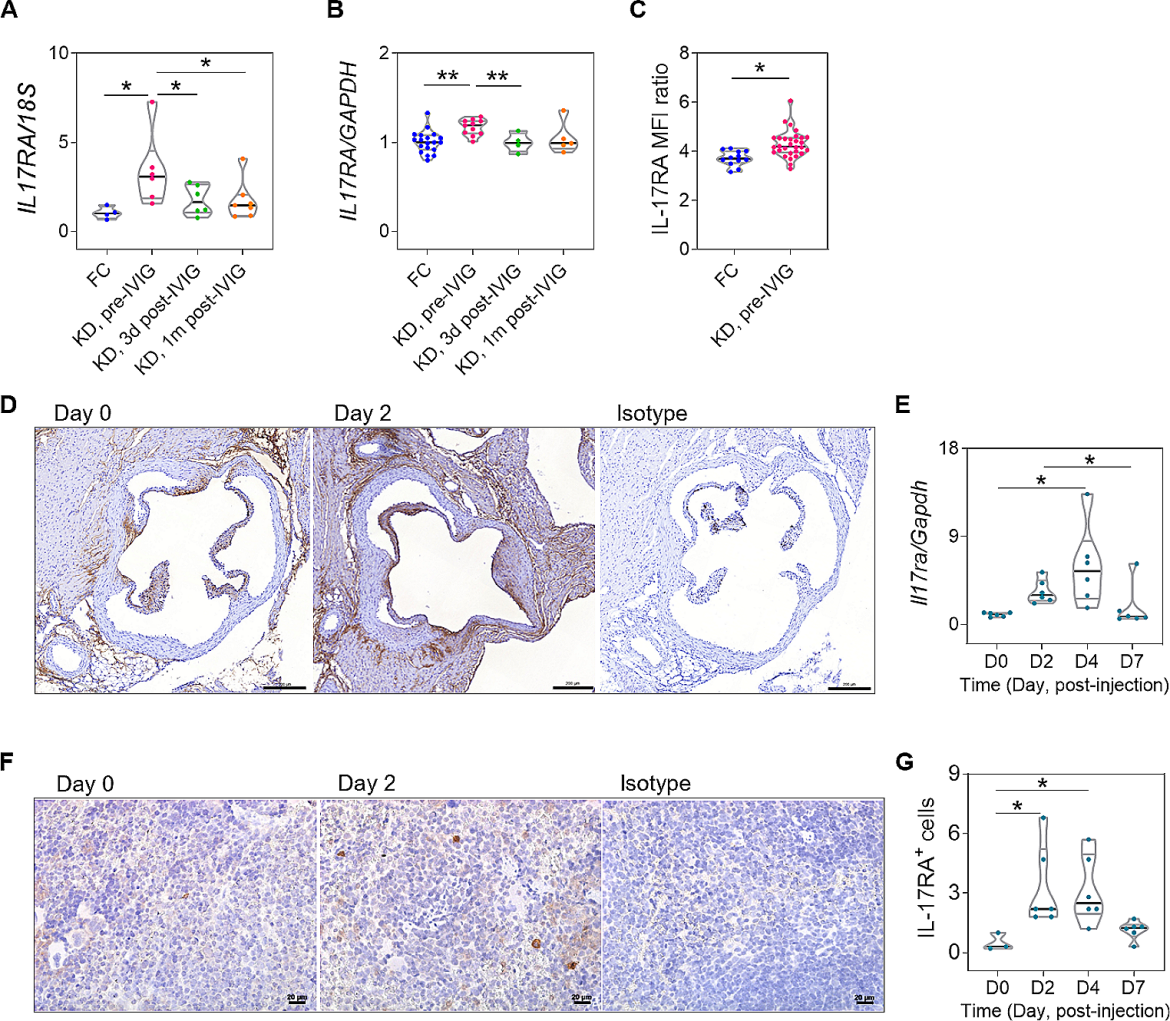
Increased IL-17RA expression in pre-IVIG KD patients and early LCWE-treated mice. **(A)** The mRNA expression levels of IL-17RA in circulating mononuclear cells and **(B)** in plasma-treated HCAECs. **(C)** The quantitative analysis for the surface expression of IL-17RA on plasma-stimulated HCAECs by flow cytometric analysis. **(D)** The representative microphotographs of immunohistochemistry study show IL-17RA protein expression of aortic roots on days 0 and 2 post-LCWE injection (scale bar: 200 μm). **(E)** The *Il17ra* mRNA levels of aortic rings were increased 2 and 4 days after LCWE injection and declined on day 7 post-injection. **(F)** The representative microphotographs of immunohistochemistry study shows that positive IL-17RA-stained cells were present in the spleens of LCWE-injected mice (scale bar: 20 μm). **(G)** The quantitative analysis reveals that the number of positive IL-17RA-stained splenocytes over 0.5 mm^2^ area were significantly increased on days 2 and 4 post-LCWE injection. Data were shown from individual experiments. *P < 0.05. IL-17RA, interleukin-17 receptor A; LCWE, *Lactobacillus casei* cell-wall extract; MFI, mean fluorescent intensity

Furthermore, we found that IL-17RA mRNA expression was significantly upregulated in the pre-IVIG circulating mononuclear cells of KD patients and significantly reduced after IVIG therapy (Fig. 3A), and was also significantly upregulated in the pre-IVIG plasma-treated HCAECs when compared to those in FC plasma-treated and 3d post-IVIG plasma-treated HCAECs (Fig. 3B). Flow cytometric analysis confirmed such IL-17RA enhancement over mononuclear cells in pre-IVIG KD patients (Figure S2) and over pre-IVIG plasma-treated HCAECs (Fig. 3C). In LCWE-stimulated mice, the enhancement of IL-17RA was localized at the valvular tissue cells, vascular wall, and myocardium near the aortic roots by immunohistochemistry study (Fig. 3D). The *Il17ra* mRNA expression levels of aortic roots were increased 2 and 4 days after LCWE stimulation and declined thereafter (Fig. 3E). Additionally, LCWE-stimulated mice had significantly higher amounts of positive IL-17RA-stained splenocytes per field of 0.5 mm^2^ area on days 2 and 4 post-injection (Fig. 3, F and G).

### Early Aortic Upregulations of IL-17A-mediated Cytokines and Chemokines, Followed by Cardiac Infiltrations of Positive Ly6G-stained Neutrophils in LCWE-induced CA

**Fig. 4:**
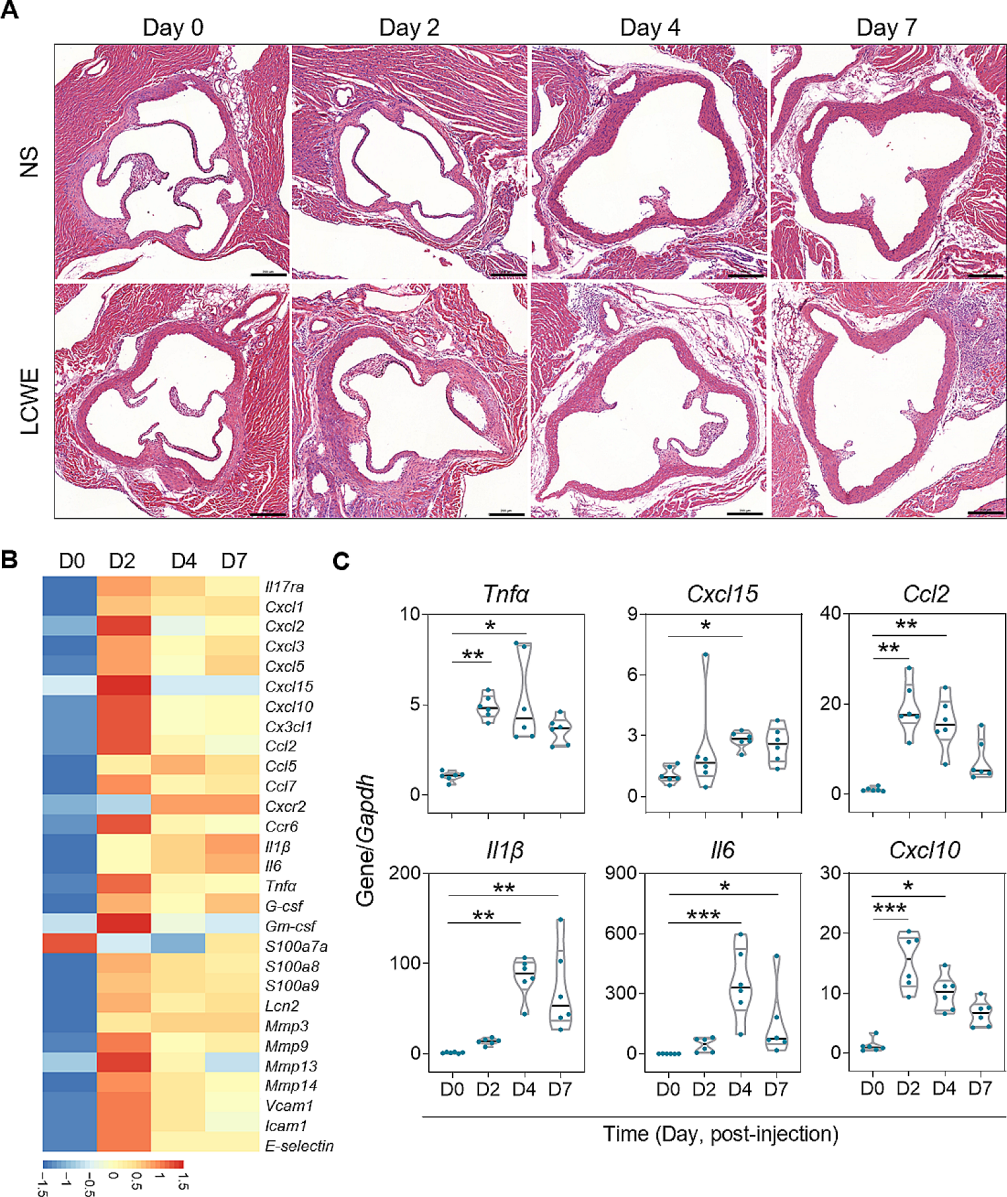
The histopathology and mRNA expression profiles of the aorta in LCWE-injected mice. **(A)** Representative histopathologic microphotographs show aortic roots and the nearby myocardium 0, 2, 4, and 7 days after injection of NS or LCWE. Coronary arteritis and aortitis were clearly observed as a great number of inflammatory infiltrates invading the adventitia and/or perivascular walls of the aorta and coronary arteries on day 7 post-LCWE injection (scale bar: 200 μm). **(B)** Heatmap shows the metal intensity of mRNA expressions from six-merged aortic roots each time point on days 2, 4, and 7 over the expression on day 0 post-LCWE injection, revealing that many genes of IL-17 A-mediated downstream signaling were upregulated. **(C)** The aortic mRNA expression levels of *Tnfα, Cxcl15* (equivalent to human *IL8*), *Ccl2*, *Il1β, Il6*, and *Cxcl10* on indicated days post-LCWE injection were validated by real-time RT-PCR. NS, normal saline; TNF-α, tumor necrosis factor-α. *P < 0.05, **P < 0.01, ***P < 0.001

**Fig. 5:**
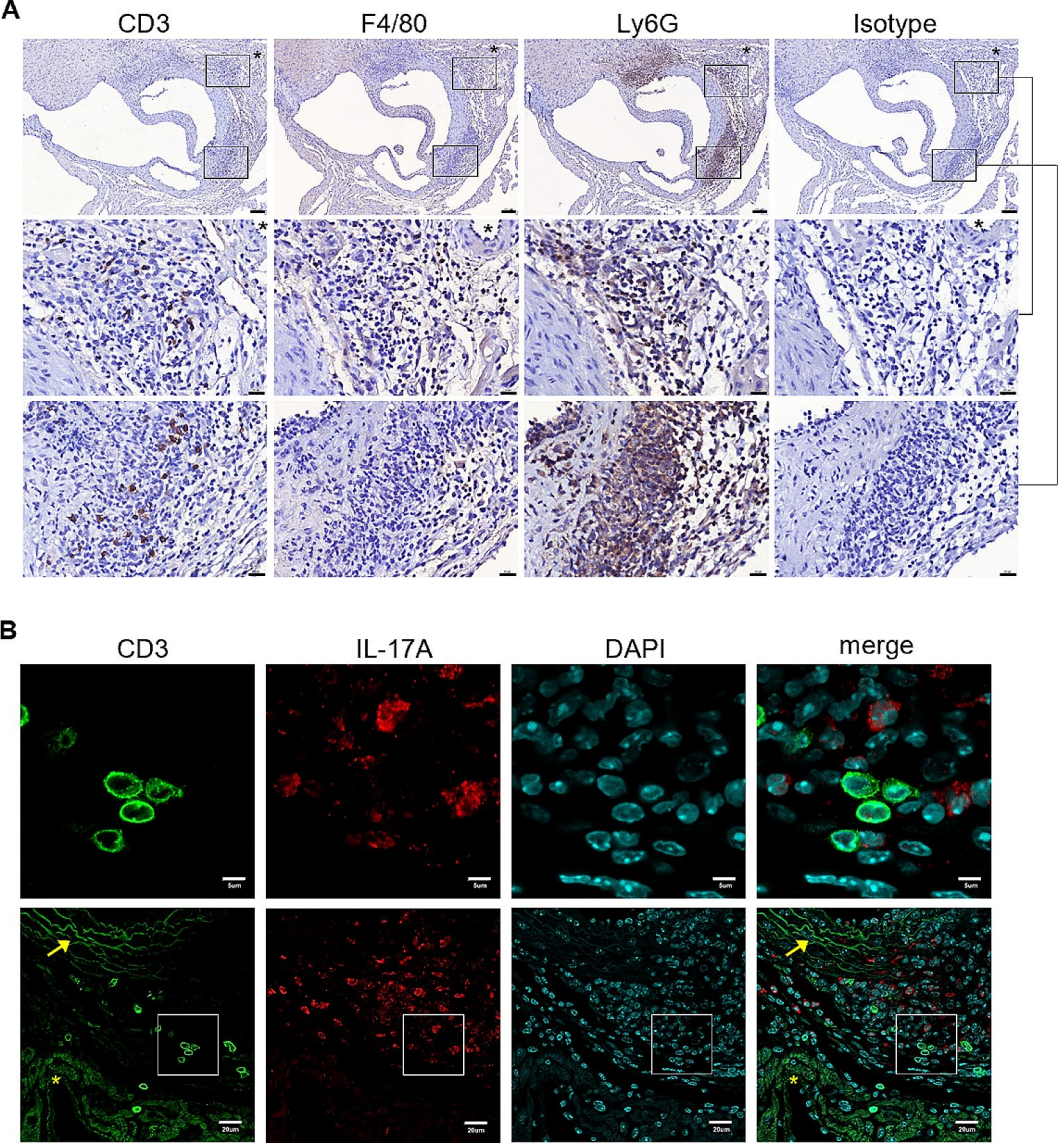
The cellular phenotypes of inflammatory infiltrates in LCWE-induced coronary arteritis and aortitis. **(A)** Representative immunohistochemistry study of serial cardiac sections shows cardiac infiltrates of positive CD3-, F4/80-, and Ly6G-stained cells around coronary arteries (asterisk insets), aortic valves, and aortic roots 7 days after LCWE injection (upper panels, scale bar: 100 μm; middle and lower panels, scale bar: 20 μm). **(B)** The representative immunofluorescence study shows positive CD3-stained cell membrane and positive IL-17 A-stained cytoplasm in such cardiac infiltrates (Arrow insets: aortic ring; asterisk insets: myocardium nearby aortic roots; upper and lower panels, scale bars: 5 and 20 μm, respectively)

To characterize the LCWE-induced CA, cardiac histopathology and the mRNA expression profile of the aortic roots were investigated. After LCWE stimulation, inflammatory infiltrates were scarcely present on day 2 post-injection and became visible from day 4 (Fig. 4A). CA and aortitis were clearly observed as inflammatory infiltrates invading the adventitia and/or vascular walls of the aorta and coronary arteries on day 7. The results of RNA-sequencing of mRNA from six-merged aortic roots on days 2, 4, and 7 post-LCWE injections revealed that many genes of IL-17A-mediated downstream signaling were upregulated (e.g., *Cxcl2, Cxcl15*, and *Ccl2*) when compared to the expression levels on day 0 (Fig. 4B). In addition to IL-17RA, the aortic mRNA expression levels of *Tnfα, Cxcl15* (equivalent to human *IL8*), *Ccl2*, *Il1β, Il6*, and *Cxcl10* were further validated by real-time RT-PCR, showing that these genes were significantly increased on days 2 and/or 4 post-LCWE injection (Fig. 4C). By immunohistochemistry study, these inflammatory infiltrates primarily composed of a large number of positive Ly6G-stained neutrophils, and some CD3-stained T cells and F4/80-stained macrophages on day 7 post-injection (Fig. 5A). Although a plenty of cells composed of either positive CD3-stained cell membrane or positive IL-17A-stained cytoplasm, dual positive CD3- and IL-17 A-stained cells were scarce among these cardiac infiltrates (Fig. 5B).

### IL-17RA-deficiency mice Developed Ameliorated LCWE-induced CA with Fewer Neutrophils

**Fig. 6:**
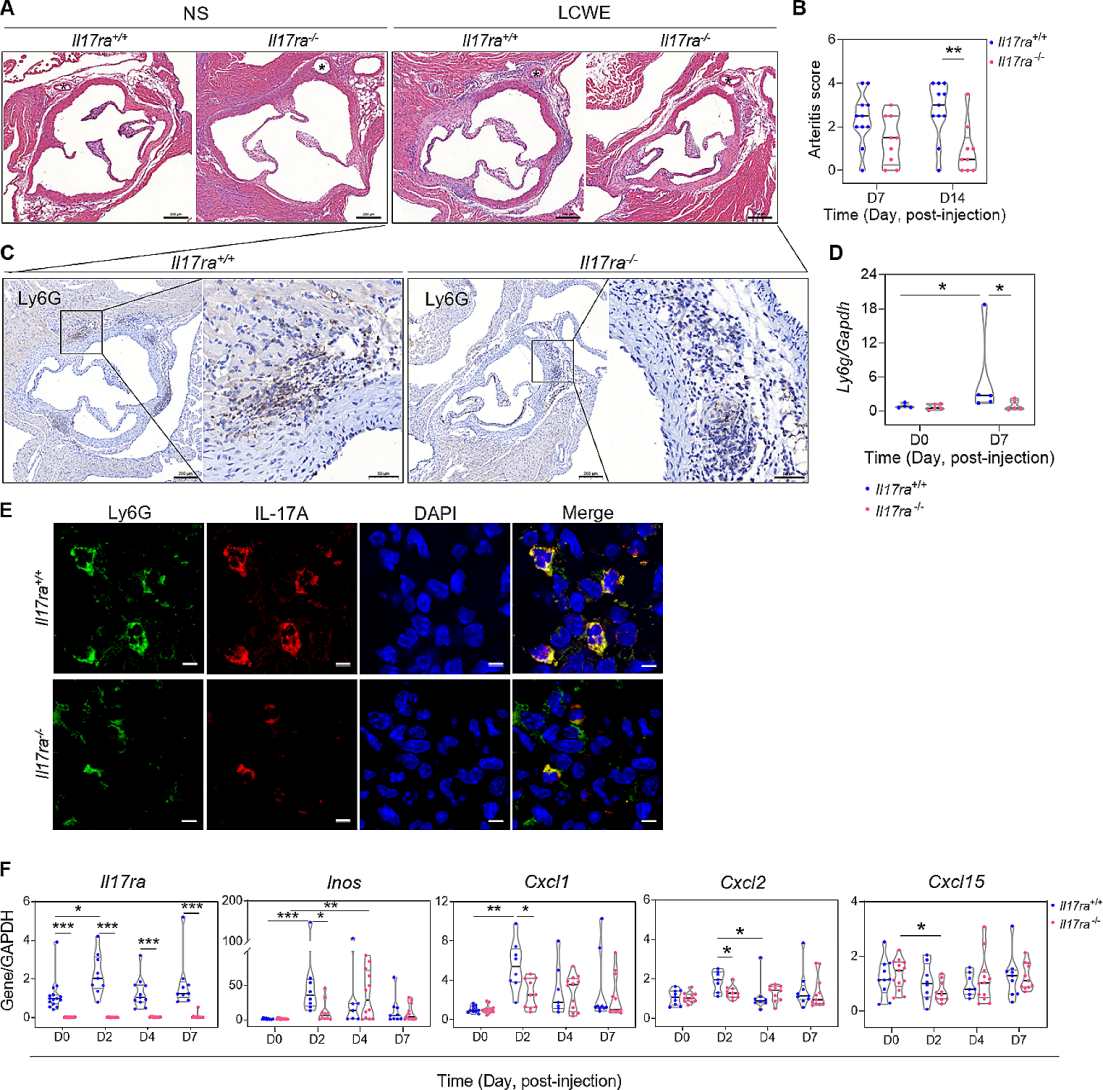
The comparison of severity and characterization in LCWE-induced arteritis between *Il17ra*^*−/−*^ mice and *Il17ra*^*+/+*^ littermates. **(A)** Representative histopathological study shows a smaller number of cardiac infiltrates (arrow insets) over the perivascular/adventitial region of the aorta and coronary artery (asterisk insets) in *Il17ra*^*−/−*^ mice than those in *Il17ra*^*+/+*^ littermates on day 7 post-injection of LCWE (scale bar: 200 μm), and **(B)** the quantitative analysis shows that the severity of LCWE-induced arteritis was ameliorated in *Il17ra*^*−/−*^ mice than *Il17ra*^*+/+*^ littermates on day 14 post-LCWE injection. **(C)** Representative immunohistochemistry study shows positive Ly6G (neutrophil marker)-stained cells in such cardiac infiltrates on day 7 post-LCWE injection (left and right panel in each strain of mouse, scale bar: 200 μm and 50 μm, respectively). **(D)** Quantitative analysis shows that the *Il17ra*^*+/+*^ littermates had significant *Ly6g* mRNA upregulation of aortic roots 7 days after LCWE injection but *Il17ra*^*−/−*^ mice had no such significant Ly6G-upregulation and had significantly lower *Ly6g* mRNA expression level than *Il17ra*^*+/+*^ littermates. **(E)** The representative immunofluorescence study shows dual positive Ly6G- and IL-17 A-stained cells in such cardiac infiltrates (scale bar: 5 μm). **(F)** The mRNA expression profiles of aortic roots after LCWE induction revealed that *Il17ra*^*−/−*^ mice had significantly lower mRNA expression levels of *Il17ra, Inos, Cxcl1*, and *Cxcl2* than *Il17ra*^*+/+*^ littermates and depressed *Cxcl15* expression on day 2 post-LCWE injection. Horizontal lines on violin plots indicate median with IQRs. *P < 0.05, **P < 0.01, ***P < 0.001

To validate the pathogenic role of IL-17A/IL-17RA in LCWE-induced arteritis, the severity of arteritis was compared between the *Il17ra*^*-/-*^ mice and *Il17ra*^*+/+*^ littermates. After LCWE induction, *Il17ra*-deficiency mice exhibited a significantly lower abundance of inflammatory infiltrates in the perivascular/adventitial region of the aorta and coronary artery and had a lower CA severity score than *Il17ra*^*+/+*^ littermates on day 14 without a significant decline in the induction rate (Fig. 6, A and B; Table S2). *Il17ra*^*-/-*^ mice had the lower amount of positive Ly6G-stained cells in such cardiac infiltrates (Fig. 6C), and had significantly lower *Ly6g* mRNA expression level of aortic roots than *Il17ra*^*+/+*^ littermates on day 7 post-injection (Fig. 6D). In addition, *Il17ra*^-/-^ mice exhibited less vascular and myocardial destruction and fibrosis at day 14 than the *Il17ra*^*+/+*^ littermates. The *Il17ra*^*+/+*^ littermates had significant Ly6G upregulation of aortic roots 7 days after LCWE injection but *Il17ra*^*-/-*^ mice had no such significant *Ly6g*-upregulation (Fig. 6D). Meanwhile, immunofluorescence study showed that positive Ly6G-stained neutrophils predominantly co-localized with positive IL-17A-stained cells in such cardiac infiltrates in both strains (Fig. 6E). Of note, only *Il17ra*^*+/+*^ littermates exhibited significant aortic upregulations and higher expression levels of *Il17ra, Inos*, *Cxcl1*, and *Cxcl2* than *Il17ra*^*-/-*^ mice on day 2 post-injection (Fig. 6F). The aortic *Cxcl15* mRNA level was even decreased in *Il17ra*^*-/-*^ mice 2 days after induction.

## Discussion

In this study, we demonstrated that KD patients especially in those with CAL had significantly higher pre-IVIG plasma level of IL17A, which was associated with concurrent maximal z-score of coronary arteries. Meanwhile, endothelial IL-17RA was involved with plasma-induced *IL8, CXCL1*, and *IL17RA* upregulations, endothelial inflammation, and neutrophil transmigration. *Il17ra-*deficiency mice developed less LCWE-induced arteritis with fewer neutrophil infiltration, absent aortic IL-17RA augmentation, and less early *Inos* and *Cxcl1* upregulations. Collectively, our data may suggest that IL-17A/IL-17RA play a significant role in the pathogenesis of KD-related CAL.

To date, limited literature dissects the pathologic role of IL-17A on KD. Current researches mainly address associations between IL-17A and KD in terms of diagnosis and IVIG responsiveness [13, 14, 32–34]. IL-17A is a cytokine and a chemokine with a double-edged sword which can either protect humans from infection or make humans diseased in several clinical scenarios [35]. It is involved in neutrophil recruitment into local tissue in defense of infection or after injury, wound healing, tissue remodeling, fibrosis, and autoimmune disorders [12]. IL-17A was initially recognized to be produced by T helper 17 (Th17) cells but many cell types are now known to be sources of it, such as CD8^+^ T cells, γδ T cells, innate lymphoid cells, and nature killer cells [12]. IL-17A functions through forming IL-17A/IL-17A homodimers and IL-17A/IL-17F heterodimers and cascades downstream pathways via binding to the dimeric IL-17RA/IL-17RC receptor complex [35]. Not surprisingly, the plasma levels of IL-17A and the concurrent level of IL-17F were modestly correlated in FC patients (*r* = 0.71, *p* < 0.001), and KD patients before (*r* = 0.55, *p* < 0.001) and 3 days (*r* = 0.47, *p* = 0.002) after IVIG. However, the significant correlation of the plasma level of IL-17F and the concurrent coronary z-scores was not observed. Meanwhile, we did not observe a significant plasma IL-17F increase or aortic *Il17f* mRNA expression early after LCWE induction in either *Il17ra*^*−/−*^*mice or Il17ra*^*+/+*^ littermates (Figure S3). It still needs further investigation to elucidate whether IL-17F contributes much less than IL-17A in mediating IL-17RA-downstream cascades for leukocyte recruitment and aortic inflammation in our model and KD-related arteritis.

Some researches found that IL-17A can directly stimulate endothelial inflammatory responses [9, 36]. Indeed, our data revealed certain degrees of positive correlations of plasma-induced endothelial inflammation genes (i.e., *ICAM1*) with the IL-17A plasma levels after 4-hour stimulation and negative correlations after 24-hour stimulation (data not shown). We also found that exogeneous IL-17A could trigger HCAECs to produce similar ranges of some mRNA expressions (i.e., *CXCL1, IL8, IL17RA*, and *CCL2*) as KD plasma did (Figure S4). The regulatory mechanisms of these genes would not be discussed herein but the reversed plasma-induced mRNA alterations and reduced transendothelial migration by in vitro IL-17RA blockade highly suggests the involvement of IL-17A/IL-17RA with endothelial inflammation in KD. Therefore, this could at least in part explain the reason why the pre-IVIG plasma level of IL-17 A correlated with maximal coronary z-scores which are taken as the clinical index for CA severity in KD. Furthermore, IL-17A mediates downstream CXCL-1, IL-8, and MIP-3α which are chemokines and cytokines involved in leukocyte recruitment and transendothelial migration of neutrophils [9], and Th17 cells [37]. Neutrophils with their secretory molecules such as proteinases are an important player in the initial damage of coronary arteries in KD, and neutrophil migration is important in early aneurysm development in human KD [38, 39]. Our data did show more prominent neutrophil infiltration than F4/80^+^ macrophage and CD3^+^ T cell infiltration in the early phase after LCWE induction in wild type mice, and the lower number of neutrophils and lower *Ly6g* mRNA expression level of aortic roots in LCWE-stimulated *Il17ra*-deficiency mice. In the autopsied KD patients, monocytes and macrophages also infiltrate into vascular wall of coronary arteries in the early KD-related CA [38]. Later, CD8^+^ lymphocytes, much more than CD4^+^ lymphocytes, with macrophages predominantly appears at the time of coronary aneurysm formation [40]. Compatible with such human histopathologic finding, the aortic MIP-3α and Th17 cells were not as much involved in the early stage of LCWE-induced CA as CXCL-1, IL-8, and positive IL-17A-stained neutrophils. Instead, Th17 cells (dual-positive CD3- and RORγ-t-stained cells) were present with higher *Il17A* mRNA expression level in the aortic roots 14 days after LCWE induction (Figure S5). Collectively, both our in vitro and animal evidence may suggest that IL-17A/IL-17RA-mediated neutrophil recruitment may play a significant role in the early stage of KD-CAL development.

A robust systemic inflammation with an intricate collaboration among multiple proinflammatory cytokines and chemokines is a feature of acute KD. In addition to IL-17A, a plenty of cytokines and chemokines relating to neutrophil activation and chemoattraction [e.g., TNF-α, IL-1β, and CXCL-10)] were markedly increased both in the systemic circulation of KD patients (Fig. 1, E-G) and at the localized aortic roots of LCWE-stimulated mice (Fig. 4). From our data, the plasma levels of TNF-α, IL-1β, and CXCL-10 in pre-IVIG KD patients were positively correlated with concurrent level of plasma IL-17A (correlation coefficient, *r* = 0.65, 0.57, and 0.31, respectively; *p* < 0.001, *p* < 0.001, and *p* = 0.01, respectively). In LCWE-injected mice, aortic *Il17ra, Tnfα, and Cxcl10* expression were provoked soon after LCWE stimulation and subsided later on; whereas, IL-1β was significantly induced slightly later, and remained upregulated on day 7 post-injection. TNF-α and IL-1β are currently thought to be ones of the key players in the immunopathogenesis of KD [3]. Several clinical trials for additional KD treatment focus on the antagonists of these related signaling pathway despite their efficacy for CAL prevention and amelioration remains controversial [41, 42]. IL-17A can induce TNF-α and IL-1β production, and synergistically collaborate with them to react to inflammation in disease [35, 43]. IL-17A and CXCL-10 in KD synergistically initiate the calcification of vascular smooth muscle cells in vitro [44]. Based on such intricate interaction and collaboration among activated immune reactions in KD, our result may indicate a potential therapeutic role of IL-17A/IL-17RA in early KD.

Of note, IL-17RA augmentation was an important finding both in KD humans and LCWE-induced mice. Possibly similar to the previous study [45], IL-17RA expression could be upregulated by IL-17A and toll-like receptors. Such local IL-17RA augmentation at aortic roots may help amplifying IL-17A/IL-17RA signal transduction (i.e., CXCL-1 and IL-8), followed by recruiting more neutrophils to aortic roots and coronary arteries. Interestingly, these recruited neutrophils were found to possibly produce IL-17A. Indeed, cumulative evidence demonstrated the pathogenic role of IL-17A-producing neutrophils in some disease models and clinical diseases [46–48]. This phenomenon maybe similar to the autocrine effect of IL-17A on neutrophil activation, which produces and responds to IL-17A [49]. Thus, a further study is mandatory to elucidate whether the ligation of endothelial or aortic *I17ra* upregulation with IL-17A-autocrine-activated neutrophils mediates important cascades for CAL formation.

The limitation of our study was the lack of in vivo evidence to prove the influence of IL-17RA upregulation and IL-17A/IL-17RA-mediated neutrophil recruitment on the severity of coronary arteritis in humans. Although this murine model of LCWE-induced CA cannot completely resemble the actual human KD, the scarcity of human cardiac specimens in the acute KD stage limits in vivo human investigation.

## Conclusions

In summary, we provided a combined human-and-animal evidence which indicated that the IL-17A/IL-17RA may play a role in mediating neutrophil recruitment to coronary arteries in the initial stage of KD-related and LCWE-induced CA, and further affecting the severity of CAL development. To develop precision medicine and to improve the global cardiovascular burden, future work is imperative to dissect the therapeutic role of IL-17A/IL-17RA signaling pathways in KD-related CAL.

### Electronic Supplementary Material

Below is the link to the electronic supplementary material.

Supplementary Material 1

## Data Availability

All data generated or analyzed and material used during this study are included in this article and its supplementary material files. Further enquiries can be directed to the corresponding author.
