# Supplementary material for: Involvement of IL-17 A/IL-17 Receptor A with Neutrophil Recruitment and the Severity of Coronary Arteritis in Kawasaki Disease
Source: J Clin Immunol. 2024 Mar 7;44(3):77. doi: 10.1007/s10875-024-01673-1 (PMC10920475; doi:10.1007/s10875-024-01673-1)
Supplement: Supplementary file 1 — Supplementary Material 1 [file 10875_2024_1673_MOESM1_ESM.docx]

Supplementary Tables

Table S1. Primer sequences of target genes and house keeping genes in human and mice.

| Human Gene | Primer sequence | Mouse Gene | Primer sequence |
| --- | --- | --- | --- |
| *18S* | Forward: GTAACCCGTTGAACCCCATT  Reverse: CCATCCAATCGGTAGTAGCG | *Gapdh* | Forward: TGCACCACCAACTGCTTAG  Reverse: GGATGCAGGGATGATGTTC |
| *GAPDH* | Forward: AATTCCATGGCACCGTCAAG  Reverse: ATCTCGCTCCTGGAAGATGG | *Cxcl1* | Forward: TGGCTGGGATTCACCTCAAG  Reverse: AGTGTGGCTATGACTTCGGTT |
| *CXCL1* | Forward: ACACTCAAGAATGGGCGGA  Reverse: TTCAGGAACAGCCACCAGTGA | *Cxcl2* | Forward: CTGAACAAAGGCAAGGCTAACT Reverse: GGCACATCAGGTACGATCCA |
| *ICAM1* | Forward: TTGAACCCCACAGTCACCTATG  Reverse: CTTCTGAGACCTCTGGCTTCGT | *Cxcl10* | Forward: ATGACGGGCCAGTGAGAATG  Reverse: TCGTGGCAATGATCTCAACAC |
| *IL8* | Forward: GAAGTTTTTGAAGAGGGCTGAGA  Reverse: TGGCATCTTCACTGATTCTTGG | *Cxcl15* | Forward: TAGGCATCTTCGTCCGTCCC  Reverse: CTGTTGCAGTAAATGGTCTCGAA |
| *IL17RA* | Forward: TTTGCCCACACCCAACAA  Reverse: CTCAAACCTGACGCACAAAC | *Il1β* | Forward: TGCCACCTTTTGACAGTGATG  Reverse: ATGTGCTGCTGCGAGATTTG |
| *INOS* | Forward: AAGTCCGACATCCAGCCGTG  Reverse: ACCACTCGCTCCAGGATACC | *Il6* | Forward: ACAAAGCCAGAGTCCTTCAGAG  Reverse: GCTTAGGCATAACGCACTAGG |
| *VEGFA* | Forward: CGAGGGCCTGGAGTGTGT  Reverse: CGCATAATCTGCATGGTGATG | *Il17a* | Forward: GGACTCTCCACCGCAATGAA  Reverse: TTTCCCTCCGCATTGACACA |
|  |  | *Il17ra* | Forward: AGTTCCCAAGCCAGTTGCAG  Reverse: TCAGCACGATGACAGATCCC |
|  |  | *Inos* | Forward: GGTGAAGGGACTGAGCTGTT  Reverse: ACGTTCTCCGTTCTCTTGCAG |
|  |  | *Ly6g* | Forward: TGCCCCTTCTCTGATGGATT |
|  |  |  | Reverse: TGCTCTTGACTTTGCTTCTGTGA |
|  |  | *Tnfα* | Forward: CAGCCTCTTCTCATTCCTGC  Reverse: CACTTGGTGGTTTGCTACGA |

Table S2. The induction rate of coronary arteritis in *Il17ra^-/-^* mice and *Il17ra^+/+^* littermates 7 (D7) and 14 days (D14) after LCWE stimulation.

|  | *Il17ra^+/+^* | *Il17ra^-/-^* | *P* |
| --- | --- | --- | --- |
| *D7* | 10/11 | 7/9 | *0.566* |
| *D14* | 10/11 | 6/9 | *0.285* |

Supplementary Figures

**
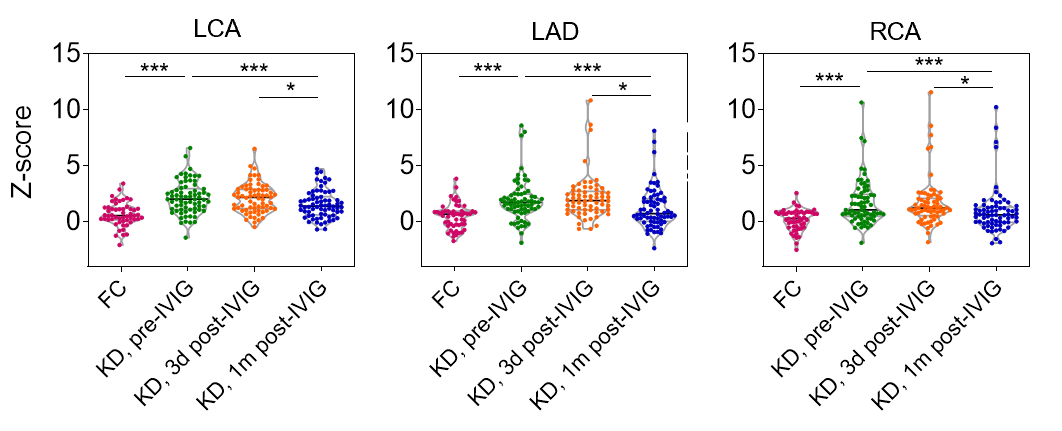
**

**Figure S1.** **The z-scores of coronary arterial diameters in patients with acute Kawasaki disease (KD) before and after intravenous immunoglobulin (IVIG) therapy.** FC, febrile control; LAD, left anterior descending coronary artery; LCA, left main coronary artery; RCA, right coronary artery. Horizontal lines on violin plots indicate median with interquartile ranges (IQRs).**P* < 0.05, ****P* < 0.001.


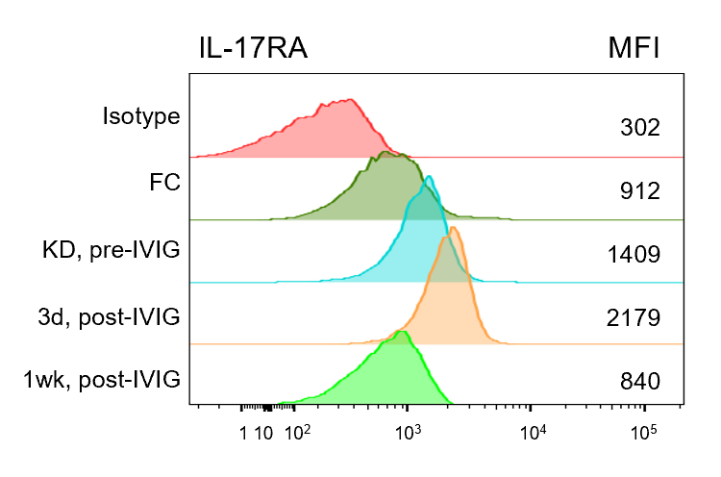


**Figure S2.** **Surface expression of IL-17RA on peripheral mononuclear cells.** Representative histograms shown the MFI (on the right side of each row) over circulating mononuclear cells from a FC and a KD patient before and after IVIG therapy by flow cytometric analysis. MFI, mean fluorescence intensity.


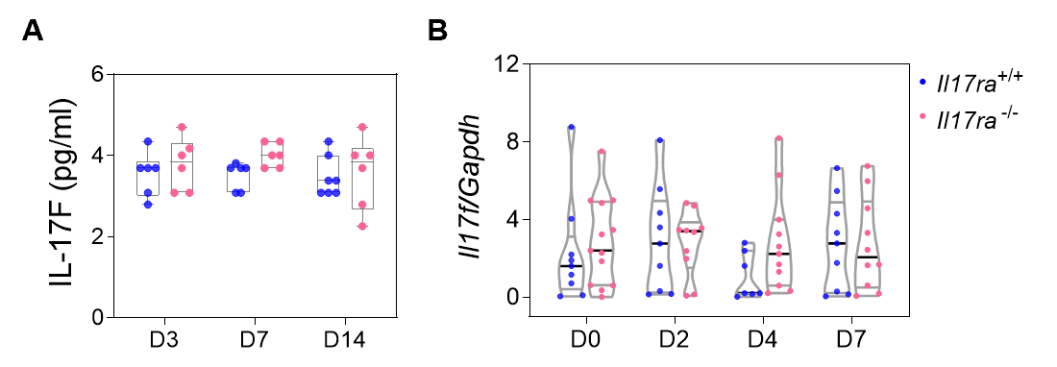


**Figure S3. The IL-17F expression in LCWE-injected mice.** (A) The plasma level of IL-17F and (B) the aortic mRNA expression level of *Il17f* in *Il17ra^-/-^ and Il17ra^+/+^* mice at indicated days after LCWE induction.


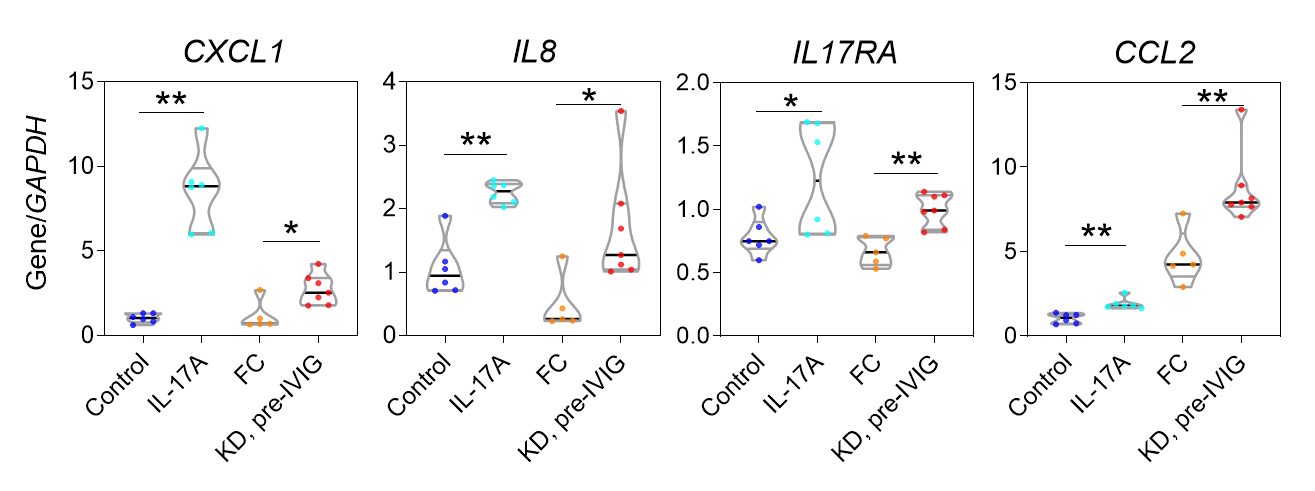


**Figure S4.** **The mRNA production by HCAECs after 24-hour stimulation with exogenous IL-17A.** The mRNA expression levels of *CXCL1, IL8, IL17RA*, and *CCL2* in HCAECs after 24-hour stimulation of recombinant IL-17A protein (at the final concentration of 10 ng/ml), and the plasma from FC and pre-IVIG KD patients.


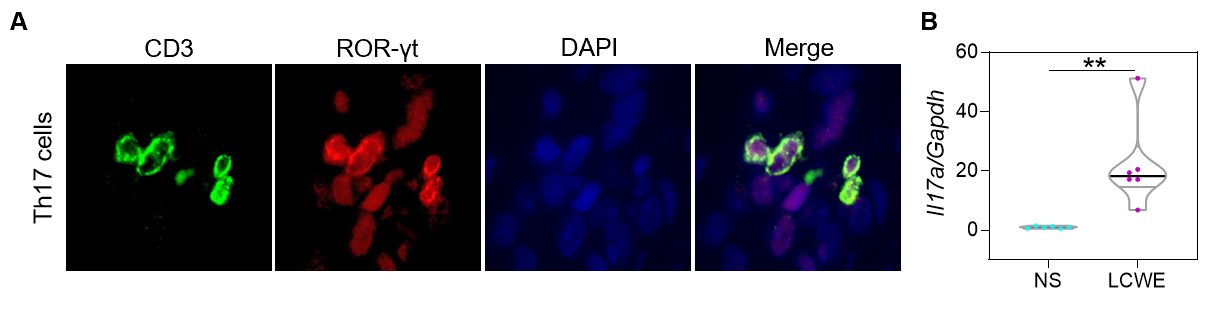


**Figure S5.** **The presence of Th17 cells and IL-17A mRNA expression levels in aortic roots.** (A) The representative immunofluorescence study shows dual positive CD3-stained cell membrane and RORγt-stained nuclei, indicative of Th17 cells, among cardiac infiltrates of aortic roots 14 days after LCWE induction in wild-type male BALB/c mice. (B) The mRNA expression levels of IL-17A in aortic roots 14 days after NS- or LCWE-stimulation.
